# Molecular Cloning and Functional Characterization of Catalase in Stress Physiology, Innate Immunity, Testicular Development, Metamorphosis, and Cryopreserved Sperm of Pacific Abalone

**DOI:** 10.3390/antiox12010109

**Published:** 2023-01-01

**Authors:** Shaharior Hossen, Zahid Parvez Sukhan, Soo Cheol Kim, Md. Abu Hanif, Il-Keun Kong, Kang Hee Kho

**Affiliations:** 1 Department of Fisheries Science, College of Fisheries and Ocean Sciences, Chonnam National University, 50 Daehak-ro, Yeosu 59626, Republic of Korea; 2 South Sea Fisheries Research Institute, National Institute of Fisheries Science, Yeosu 59780, Republic of Korea; 3 Department of Animal Science, Division of Applied Life Science (BK21 Four), Gyeongsang National University, Jinju 52828, Republic of Korea

**Keywords:** catalase, innate immunity, stress physiology, starvation, metamorphosis, cryopreserved sperm, Pacific abalone

## Abstract

Catalase is a crucial enzyme of the antioxidant defense system responsible for the maintenance of cellular redox homeostasis. The aim of the present study was to evaluate the molecular regulation of catalase (*Hdh-CAT*) in stress physiology, innate immunity, testicular development, metamorphosis, and cryopreserved sperm of Pacific abalone. *Hdh-CAT* gene was cloned from the digestive gland (DG) of Pacific abalone. The 2894 bp sequence of *Hdh-CAT* had an open reading frame of 1506 bp encoding 501 deduced amino acids. Fluorescence *in situ* hybridization confirmed *Hdh-CAT* localization in the digestive tubules of the DG. *Hdh-CAT* was induced by different types of stress including thermal stress, H_2_O_2_ induction, and starvation. Immune challenges with *Vibrio*, lipopolysaccharides, and polyinosinic–polycytidylic acid sodium salt also upregulated *Hdh-CAT* mRNA expression and catalase activity. *Hdh-CAT* responded to cadmium induced-toxicity by increasing mRNA expression and catalase activity. Elevated seasonal temperature also altered *Hdh-CAT* mRNA expression. *Hdh-CAT* mRNA expression was relatively higher at the trochophore larvae stage of metamorphosis. Cryopreserved sperm showed significantly lower *Hdh-CAT* mRNA expression levels compared with fresh sperm. *Hdh-CAT* mRNA expression showed a relationship with the production of ROS. These results suggest that *Hdh-CAT* might play a role in stress physiology, innate immunity, testicular development, metamorphosis, and sperm cryo-tolerance of Pacific abalone.

## 1. Introduction

Catalase (CAT) is the most frequently used biomarker of oxidative stress in animals of aquatic environments [1,2]. Catalase is a vital antioxidant enzyme that can balance the redox system by regulating antioxidant defenses against oxidative stress [3,4]. This antioxidant enzyme responds to the adverse effects of environmental pollution on organisms [1]. CAT is a part of the reactive oxygen species (ROS) network and plays a central role in balancing hydrogen peroxide (H_2_O_2_) levels in cells [5,6,7]. The key biological mechanism of CAT is to scavenge the excessive level of ROS by directly breaking down H_2_O_2_ into water and oxygen [8,9,10,11]. H_2_O_2_ is a highly reactive ROS [12] and a secondary key performer of mitochondrial ROS production [13]. Excessive H_2_O_2_ lead to infertility whereas CAT restored fertility [14]. H_2_O_2_ challenges induce CAT expression in mollusks [3,15]. Whereas heat stress induces H_2_O_2_ production [16,17] that ultimately increases CAT expression [18,19,20]. Heat stress also induces CAT activity and increases its mRNA expression in a marine mollusk, *Scapharca subcrenata* [21]. Cold stress imbalances CAT activity and expression in scallop, *Chlamys nobilis* [4]. CAT neutralizes the effects of ROS production during starvation [22]. Starvation induces CAT activity and mRNA expression in fish [22,23].

CAT also plays an important role in the innate host defense mechanism against environmental stress and pathogen infection [24]. CAT is a reliable biomarker for detecting a variety of pollutants [25]. It can defend against metal-oriented stress [26]. Cadmium (Cd) is a bioaccumulation environmental pollutant able to induce ROS [27] and finally damage the physiological function of organs [26]. Antioxidant activity of CAT has been previously detected in Cd exposed-aquatic organisms [26]. *Vibrios* are categorized as the main infected pathogenic bacteria of abalone [28]. Bacterial challenges of disk abalone can induce CAT expression [28], which proves that CAT might play a role in the immune defense mechanism against pathogens [15]. Viral challenge also alters *CAT* mRNA expression in disc abalone [28]. 

The antioxidant defense system of CAT is altered seasonally in response to several types of environmental factors [29,30]. Elevated temperature modulates the activity and expression of CAT in the gill and digestive glands of American oyster [31]. 

CAT also plays a potential role in sperm physiology, which is essential for normal sperm function and spermatogenesis. CAT is a molecular marker to determine fertility [14]. Higher activity and abundance of CAT have been found in aquatic organisms during early embryogenesis [32,33,34].

Cryopreservation of sperm is responsible for excessive ROS generation that causes an imbalance of the natural antioxidant defense system of sperm [35]. CAT activity and mRNA abundance have been found to be antioxidant indicators of cryopreserved sperm and ovary [35,36].

Abalone are high-priced marine bioresources found worldwide in tropical and temperate waters [37]. Abalone are key components of the marine ecosystem as well as the aquaculture industry [38]. Dynamic environmental and pathogenic conditions of the intertidal zone create various environmental stresses [39]. These adverse environmental conditions lead to a decrease in the survivability of abalone [38,39]. Abalone are vital bio-indicators of the marine ecosystem [39] due to their innate responses against viruses, bacteria, and toxic substances [28]. Of various abalone species, Pacific abalone *Haliotis discus hannai* is the most demanded seafood in Korea, Taiwan, China, and Japan, because it contains bioactive molecules [40]. It is also known as the “emperor of shellfish” in Korea [41] and recognized worldwide as the “soft gold” of the Ocean [42,43]. Among abalone, to date, CAT has been reported in disk abalone, *H. discus discus* [3]. CAT is highly expressed in the DG and gill of disk abalone, *H. discus discus* [3]. The DG, gill, and blood are important immune organs of abalone [44]. Moreover, the DG (hepatopancreas) is the main metabolic organ of Pacific abalone. The DG act as the major site for the accumulation of bacteria and toxic metals, playing a vital role against oxidative stress by eliminating excessive ROS [44]. However, molecular characterization of CAT in Pacific abalone is unknown. Thus, the objective of this study was to obtain the full-length cDNA of CAT from Pacific abalone and apply this sequence to understand the molecular characterization of CAT in stress physiology, immune response, elevated seasonal temperature, testicular development, metamorphosis, and cryopreserved sperm of Pacific abalone. Furthermore, the localization of CAT was determined using fluorescence *in situ* hybridization.

## 2. Materials and Methods

### 2.1. Ethics Statement

The experimental protocols were approved by the Institutional Animal Care and Use Committee (IACUC) of Chonnam National University (CNU IACUC-YS-2020-5). All experiments were conducted following the Guidelines for the Care and Use of Laboratory Animals of the National Institutes of Health.

### 2.2. Experimental Abalone Collection

Biologically matured three years old Pacific abalone were collected from sea cages of Wando-gun, Republic of Korea. A total of 512 Pacific abalone having a mean body weight of 134.8 ± 14.3 g were used in the experiments.

### 2.3. Tissue Collection for Gene Cloning

Abalone (*n* = 10) were euthanized for collecting digestive gland (DG) tissue samples. The samples were washed using 0.1M phosphate-buffered saline (PBS) and subsequently snap-frozen in liquid nitrogen (LN_2_). Samples were then stored at −80 °C until total ribonucleic acid (tRNA) extraction.

### 2.4. Tissue Collection of Different Organs of Pacific Abalone

Abalone including digestive gland (DG), testis (TE), ovary (OV), muscle (MUS), mantle (MA), gill (G), heart (HRT), hemocyte (HCY), cerebral ganglion (CG), and pleuropedal ganglion (PPG) were collected to check the tissue distribution of catalase. 

### 2.5. Fluorescence In Situ Hybridization (FISH) to Localize Hdh-CAT in Digestive Gland Tissue of Pacific Abalone

#### 2.5.1. Tissue Collection for Fluorescence In Situ Hybridization

Abalone digestive gland tissues (*n* = 6) were collected after performing anesthesia with 5% MgCl_2_. Samples were washed with pre-chilled 0.1 M PBS and fixed with 4% paraformaldehyde (PFA) for in situ hybridization.

#### 2.5.2. Riboprobe Synthesis 

Riboprobe synthesis was performed using the method described previously [45], with slight modifications. Briefly, sense and antisense mRNA probes were prepared from 714 bp fragments of the catalase domain of *Hdh-CAT* that had been amplified using a pair of primers (Table S1). Sense and anti-sense riboprobes were separately labeled with fluorescein-12-UTP (Roche, Grenzach-Wyhlen, Germany) using SP6 or T7 RNA polymerase (Promega, Medison, WI, USA). A total 20 μL working solution including 1 μg plasmid DNA, 2.0 μL SP6 or T7 RNA polymerase, 4 μL 5X optimized transcription buffer, 2 μL DTT (100 mM), 2 μL RNase inhibitor, 2 μL fluorescein RNA labeling mix, and 7 μL RNase-free water was prepared and incubated for 2 h at 37 °C. Then, the samples were digested with 2.0 μL DNase I and 0.5 μL RNase-OUT at 37 °C for 15 min. Finally, riboprobes were purified using ethanol precipitation with 1 μL yeast tRNA (10 mg/mL, Sigma-Aldrich, St. Louis, MO, USA). The probes were stored at −80 °C until use.

#### 2.5.3. Preparation of Tissue Sections

Paraformaldehyde-fixed digestive gland samples of Pacific abalone were infiltrated using a 30% sucrose solution. Tissue samples were embedded in optimum cutting temperature compounds (FSC 222, Leica Biosystems, Wetzlar, Germany). Transverse-oriented tissues were then sectioned at 8 μM in thickness using a cryostat device (CM 3050, Leica, Wetzlar, Germany) and mounted on electrostatically charged glass slides (SuperFrost Plus, Radnor, PA, USA). Slides were air-dried for 30 min and stored at −20 °C until use. Consecutive slides were prepared with alternative sections for the hybridization of *Hdh-CAT* mRNA using sense and antisense riboprobes.

#### 2.5.4. Fluorescence In Situ Hybridization (FISH)

FISH was performed following a previously described protocol [45] and DIG in situ hybridization manual with minor modifications. Briefly, a total of 50 mL hybridization buffer (HB) was prepared using deionized formamide (25 mL), 20X saline sodium citrate (SSC, 12.5 mL), 0.1% Tween-20 (0.5 mL), 1M citric acid (0.46 mL), and DEPC-treated water (11.54 mL). Yeast tRNA was mixed with HB at a ratio of 1:9 to prepare HB mix. Digestive gland tissue sections were prehybridized with HB mix for 2 h at 65 °C, followed by hybridization with fluorescein-12-UTP-labeled RNA probe (200 ng/mL, diluted with HB mix) at 65 °C overnight. Samples were subsequently washed with degraded series (75%, 50%, and 25% volume) of hybridization mix with 2X SSC for 10 min each at 65 °C. Samples were then washed with SSC (2X and 0.2X) for 15 min. Then, the tissue sections were sequentially cleaned with degraded series of 0.2X SSC (75%, 50%, and 25% volume) mixed with PBST for 5 min and washed with PBST for 5 min. Tissue sections were then incubated with calf serum (10%) for 1 h at 25 °C and then incorporated with Fab fragments antibody (anti-digoxigenin-fluorescein) for 1 h at 25 °C. Samples were then washed three times (10 min each) with PBST, followed by three times of wash with alkaline-tris buffer for 5 min each. Finally, hybridized tissue samples were counterstained and mounted using VECTASHIELD^®^ antifade mounting medium with DAPI (Vector Laboratories, Burlingame, CA, USA). Fluorescence catalase signals were visualized and captured using a confocal microscope with Airyscan2 (LSM 900, ZEISS, Oberkochen, Germany). Images were processed on ZEISS ZEN 3.2 (Blue 268 edition, Oberkochen, Germany) software.

### 2.6. Thermal Stress Experiments

#### 2.6.1. Cold Stress of Pacific Abalone at 15 °C

Cold temperature stress (15 °C) was carried out by transferring abalone into experimental tanks. Water temperature was reduced from 20 °C to 15 °C with a temperature reduction rate of 1 °C/h until it reached 15 °C. Cold temperature (15 °C) was maintained using an electric cooling unit (SunCool, DA-3000C, DAEIL, Busan, Republic of Korea). Abalone reared at 20 °C were sampled as a control, and the cold stressed at 15 °C were sampled at 1 h, 6 h, 12 h, 24 h, 48 h, and 72 h, respectively. Before sampling, abalone were anesthetized with 5% MgCl_2_. Digestive gland and gill tissue samples (*n* = 6) were collected and washed with 0.1 M PBS. Hemolymph samples were collected and plasma samples were separated for catalase activity assay. Samples were snap-frozen in LN_2_ and stored at −80 °C until tRNA extraction and catalase activity assay.

#### 2.6.2. Heat Stress of Pacific Abalone at 25 °C and 30 °C

Abalone were transferred into experimental tanks and acclimatized before conducting the thermal stress experiment. Water temperature was gradually increased from 20 °C to 25 °C and 20 °C to 30 °C at 1 °C/h until it reached 25 °C or 30 °C. Abalone reared at 20 °C were used as control, and the heat-stressed abalone at 25 °C and 25 °C were sampled at 1 h, 6 h, 12 h, 24 h, 48 h, and 72 h. Digestive gland and gill tissue samples (*n* = 6) were collected and washed with 0.1 M PBS. Hemolymph samples were collected and plasma samples were separated for catalase activity assay. Samples were snap-frozen in LN_2_ and stored at −80 °C until tRNA extraction and catalase activity assay.

### 2.7. Seasonal Sample Collection

To detect *Hdh-CAT* mRNA levels in seasonal samples, abalone were collected in different seasons including winter, summer, autumn, and spring (Wando-gun, Republic of Korea), and transported to Molecular Physiology Laboratory, Chonnam National University. After anesthetizing, digestive gland and gill sample (*n* = 10) were collected and stored at −80 °C until tRNA extraction.

### 2.8. H_2_O_2_ Induction Treatments 

Abalone were transferred into experimental tanks and acclimatized one week before the H_2_O_2_ induction experiment. Abalone were reared in seawater-supplied tanks with continuous aeration. Abalones (*n* = 6) were intramuscularly injected with 50 μL (0.3 mg/mL) of H_2_O_2_ and an equal volume of PBS was injected into control abalone. Digestive gland and gill samples (*n* = 6) were collected at 3 h, 6 h, 12 h, 24 h, and 48 h. A blood sample was collected and separated the plasma for catalase activity. Samples were immediately snap-frozen in LN_2_ and stored at −80 °C until tRNA extraction and catalase activity assay.

### 2.9. CdCl_2_ Exposed Treatments 

Acclimatized abalone were transferred to a 50 L aquarium to conduct CdCl_2_ (Sigma-Aldrich, St. Louis, MO, USA) exposed treatments of Pacific abalone. The Cd^2+^ at concentrations of 1.5 mgL^−1^, 3 mgL^−1^, 6 mgL^−1^, and 12 mgL^−1^ were selected to conduct this experiment. Abalone were exposed to different concentrations of Cd^2+^ and the samples (digestive gland and gill) were collected at 3 h, 6 h, 12 h, 24 h, and 48 h. A blood sample was collected and separated the plasma for catalase activity assay. Samples were immediately snap-frozen in LN_2_ and stored at −80 °C until tRNA extraction and catalase activity assay.

### 2.10. Tissue Collection of Starved Pacific Abalone

Experimental abalone were starved for 21 days and re-fed for 7 days. Abalone were reared in the experimental aquarium (50 L). Digestive gland, gill, and hemolymph samples (*n* = 6) were collected from 7 days (st-7 d), 14 days (st-14 d), 21 days (st-21 d) starved, and 7 days re-fed (ref-7 d) abalones. Samples were immediately snap-frozen in LN_2_ and stored at −80 °C until tRNA extraction and catalase activity assay. 

### 2.11. Immune Challenges of Pacific Abalone to Check the Hdh-CAT Activity and mRNA Abundance

Lipopolysaccharides from *Escherichia coli* O55:B5 (LPS, Sigma-Aldrich, Saint Louis, MO, USA), polyinosinic–polycytidylic acid sodium salt (PIC, Sigma-Aldrich, Saint Louis, MO, USA), and *Vibrio parahaemolyticus* (ATCC 17802, Koram Biotech Corp., Seoul, Republic of Korea) were used to conduct the immune challenge experiments. LPS and PIC were separately injected into the adductor muscle at a concentration of 10 µg/g-BW. *V. parahaemolyticus* were cultured in Luria-Bertani broth (Becton, Dickinson and Company, Sparks, MD, USA) and injected in the adductor muscle at a previously recommended dose [46]. Control abalone were injected with an equal volume of PBS. Digestive gland, gill, and hemolymph samples (*n* = 6) were collected 3 h, 6 h, 12 h, 24 h, and 48 h after the injection. Samples were immediately snap-frozen in LN_2_ and stored at −80 °C until tRNA extraction and catalase activity assay.

### 2.12. Tissue Collection of Testicular Developmental Stages

Testis tissue samples (*n* = 10) were collected during gonadal development stages including the degenerative stage (DS), active stage (AS), ripening stage (RS), and spent stage (SS) as described previously [47]. 

### 2.13. Sperm Cryopreservation of Pacific Abalone

To determine *Hdh-CAT* mRNA levels in cryopreserved sperm samples, Pacific abalone sperm samples were cryopreserved according to the protocol described previously [48]. Different types of penetrating cryoprotectants namely 8% dimethyl sulfoxide (8% DMSO), 2% glycerol (2% GLY), 8% ethylene glycol (8% EG), 6% propylene glycol (6% PG), and 2% methanol (2% MeOH), with or without antifreeze protein (AFPIII) were used as reported previously [41,48]. Briefly, stripped sperm over 90% motility was used for sperm cryopreservation. Sperm were diluted with filtered seawater at a ratio of 1:10 (*v*/*v*). Diluted sperm samples were then mixed with cryoprotectant solution (8% DMSO + AFPIII, 2% GLY + AFPIII, 8% EG + AFPIII, 6% PG + AFPIII, and 2% MeOH + AFPIII) at a ratio of 1:1 (*v*/*v*). Sperm were then transferred into 0.50 mL straws. The straws were then placed at a 5 cm rack height of LN_2_ for 10 min and subsequently submerged into LN2. Straws were thawed at 60 °C for 5 s in a seawater bath (JISICO Lab & Scientific Instrument, Seoul, Republic of Korea). After thawing, the sperm samples were prepared as previously described [48]. 

### 2.14. Samples Collection of Metamorphosis Stages of Pacific Abalone

Experimental samples during metamorphosis stages including unfertilized egg (UF), 2-cell (2-CL), 4-cell (4-CL), morula (MO), blastula (BL), trochophore (TR), veliger (VEL), shell formed (SHF), and juvenile (JUV) were collected during peak reproductive season (May). Samples were immediately snap-frozen in LN_2_ and stored at −80 °C until tRNA extraction and catalase activity.

### 2.15. Total RNA Extraction and cDNA Synthesis

Total RNA of all collected samples was extracted using an ISOSPIN Cell & Tissue RNA kit (Nippon Gene, Tokyo, Japan). Total RNA concentration was measured using a spectrophotometer (Nanodrop ACTGene ASP-2680, Piscataway, NJ, USA). cDNA synthesis was accomplished by reverse transcribing the tRNA using a Superscript^®^III First-Strand synthesis kit (Invitrogen, Carlsbad, CA, USA) following the manufacturer’s procedures.

### 2.16. Molecular Cloning and Sequencing of Catalase Gene (Hdh-CAT) in Haliotis discus hannai

#### 2.16.1. Cloning of a Partial *Hdh-CAT* Sequence

To obtain a partial *Hdh-CAT* sequence, cDNA synthesized from the digestive gland was reverse transcribed using reverse transcription (RT) primers (Table S1) designed from known catalase sequence of *H. discus discus* (GenBank accession no. DQ821496.1). Reverse transcription polymerase chain (RT-PCR) reaction mixture (20 μL) was prepared using a 1 μL cDNA template, 1 μL of each sense and antisense primers (20 pmol), 4 μL of HF buffer, 2 μL of dNTP mix, 0.5 μL of DNA polymerase, and 10.5 μL sterile distilled water (dH_2_O). The RT-PCR was conducted in a thermal cycler with the following conditions: an initial denaturation at 94 °C for 3 min; 35 cycles of denaturation at 94 °C for 30 s; annealing at 58 °C for 30 s; and extension at 72 °C for 30 s; with a final dissociation step of 5 min at 72 °C. The PCR products were separated on 1.2% agarose gel and purified using a gel extraction kit (Promega, Madison, WI, USA). Purified DNA was ligated to a pTOP Blunt V2 vector (Enzynomics, Daejeon, Republic of Korea) and transformed into Escherichia coli DHα competent cells (Enzynomics, Daejeon, Republic of Korea). Plasmid DNA was extracted from positive clones using a Hybrid-QTM Plasmid Rapidprep mini kit (GeneAll, Seoul, Republic of Korea). Sequencing was performed using the Macrogen Online Sequencing System (Macrogen, Seoul, Republic of Korea).

#### 2.16.2. Cloning of the Full-Length *Hdh-CAT* Sequence

To obtain full-length *Hdh-CAT* sequence, gene-specific primers (GSP) were designed (Table S1) from a partial sequence, including a 15 bp overlap with the 5′ end of GSP sequences. Rapid amplification of cDNA ends (RACE) PCR was conducted using 50 μL volume of PCR mixture containing 2.5 μL of first-strand cDNA from the digestive gland, 5 μL of universal primer mix, 25 μL of SeqAMP buffer, 1 μL of SeqAMP DNA polymerase, and 15.5 μL pf PCR grade water. Touchdown PCR was performed with 25 cycles for rapid amplification of 3′ cDNA ends (3′-RACE) and 5′-RACE PCR following the manufacturer’s protocol. PCR products were purified from 1.2 % agarose gel using a NucleoSpin^®^ Gel and PCR Clean-up kit (MARCHERY-NAGEL GmbH & Co. KG, Düren, Germany). Purified products were cloned into a linearized pRACE vector (Clontech Laboratories, Inc., Mountain View, CA, USA) and transformed into Stellar competent cells. Plasmid DNA extraction and sequencing were performed as the method described in the “Cloning of partial *Hdh-CAT* sequence” section. Finally, the sequences were combined by overlapping with the initial cloned fragment to obtain the full-length sequence of *Hdh-CAT*. 

### 2.17. Sequence Analysis

A series of bioinformatics tools were used for the sequence analysis of Hdh-CAT. The protein sequence of Hdh-CAT was predicted using ORFfinder, a National Center for Biotechnology Information (NCBI) tool (https://www.ncbi.nlm.nih.gov/orffinder/, accessed on 8 November 2021). The protein homology of Hdh-CAT was performed using the Basic Local Alignment Search Tool (BLASTP) (https://blast.ncbi.nlm.nih.gov/Blast.cgi, accessed on 8 November 2021). Identification and annotation of CAT domain architecture were performed using the simple modular architecture research tool (SMART) (http://smart.embl-heidelberg.de/, accessed on 8 November 2021). The molecular weight and theoretical isoelectric point (PI) of Hdh-CAT were determined using ProtParam (https://web.expasy.org/protparam/, accessed on 4 February 2022). Motif scan analysis was performed using a motif scan program (https://myhits.sib.swiss/cgi-bin/motif_scan, accessed on 4 February 2022). The conserved motifs in the Hdh-CAT amino acid sequence were analyzed using Multiple Em for Motif Elicitation (MEME) online tools (http://meme-suite.org/tools/meme, accessed on 4 February 2022). Multiple protein sequences were aligned using Clustal Omega (https://www.ebi.ac.uk/Tools/msa/clustalo/, accessed on 5 February 2022). The multiple sequence alignments were edited and visualized using Jalview Java alignment editor version 2.11.1.7. Gene ontology of Hdh-CAT was predicted using Contact-guided Iterative Threading ASSEmbly Refinement (C-I-TASSER) protein structure prediction server (https://zhanggroup.org/C-I-TASSER/, accessed on 2 February 2022).

### 2.18. Phylogenetic Analysis

Catalase protein sequences of different organisms were retrieved from the NCBI database and selected to construct the phylogenetic tree. Protein sequences were aligned using the Clustal Omega program. Phylogenetic and molecular evolutionary analyses were performed using MEGA 11 (https://www.megasoftware.net/, accessed on 5 February 2022) with the neighbor-joining algorithm following 1000 bootstrap replicates. 

### 2.19. The Three-Dimensional Protein Structure of Hdh-CAT

The three-dimensional (3D) protein structure of Hdh-CAT was generated using an online protein structure and function prediction program, Iterative Threading ASSEmbly Refinement (I-TASSER; https://zhanggroup.org/I-TASSER/, accessed on 4 February 2022). Chimera software (https://www.cgl.ucsf.edu/chimera/, accessed on 6 February 2022) was used to analyze and visualize the predicted 3D structure of Hdh-CAT. Heme binding and NADPH binding sites were detected using an online program, SWISS-MODEL (https://swissmodel.expasy.org/, accessed on 7 February 2022). 

### 2.20. Quantitative Real-Time PCR (qRT-PCR) Analysis

qRT-PCR analysis was performed to quantify *Hdh-CAT* mRNA expression in different types of tissue samples. qRT-PCR was conducted on a LightCycler^®^ 96 system (Roche, Grenzach-Wyhlen, Germany) using a 2× qPCRBIO SyGreen Mix Lo-Rox kit (PCR Biosystems, Ltd., London, UK) as described previously [49]. Gene-specific sense and antisense primers (Table S1) were designed to quantify *Hdh-CAT* mRNA in different tissues. PCR was performed using a 20 μL volume of reaction mix containing 1 μL of cDNA, 1 μL of each sense and antisense primer, 10 μL of SyGreen Mix, and 7 μL of dH_2_O. The following melting temperature was used as default settings: 95 °C for 10 s, 65 °C for 1 min, and 97 °C for 1 min. The relative *Hdh-CAT* mRNA expression was quantified using the 2^−ΔΔct^ method [50]. Expression levels were normalized by amplifying a housekeeping Pacific abalone *β-actin* gene.

### 2.21. Catalase Activity 

Catalase activity in hemolymph samples was determined using a catalase colorimetric activity kit (Invitrogen, Frederick, MD, USA) according to the manufacturer’s instructions. Catalase concentration (U/mL) at 560 nm was measured (*n* = 3) using an Epoch^TM^ Microplate Spectrophotometer (Epoch 2, BioTek, Winooski, VT, USA).

### 2.22. Fluorescent Technique to Detect ROS in DG Tissue Samples of Pacific Abalone

Thermal stress (15 °C and 30 °C), H_2_O_2_ induced, starved, Cd-exposed (12 mgL^−1^) and *V. parahaemolyticus*-challenged digestive glands were used to conduct this experiment. Tissue samples were selected based on higher *Hdh-CAT* mRNA abundance levels (Heat stressed abalone: 48 h; starved abalone: st-21 d; H_2_O_2_-induced abalone: 12 h; vibrio challenged abalone: 12 h; and Cadmium-exposed abalone: 6 h), and the samples of the experimental peak time point (Heat stressed abalone: 72 h; re-fed abalone: ref-7 d; H_2_O_2_-induced abalone: 48 h; vibrio-challenged abalone: 48 h; and Cadmium-exposed abalone: 48 h). Reactive oxygen species (ROS: O_2_^•−^ production) were detected using a DHE (dihydroethidium) assay kit (Invitrogen Molecular Probes, Eugene, OR, USA) according to the method described previously [51], with minor modifications. Briefly, digestive gland tissue samples were homogenized and washed using 0.1M PBS. Tissue samples were stained with 10 µM DHE and incubated for 30 min at 10 °C in the dark. DHE-stained cells were visualized under a fluorescence microscope (red filter: 510–560 nm, Eclipse E600, Nikon, Tokyo, Japan) with a 20× objective lens. Gray values of 200 cells in each treatment were measured using ImageJ software version 1.8.0_172 (https://imagej.nih.gov/ij/download.html, accessed on 13 April 2022). 

### 2.23. Statistical Analysis 

Changes of *Hdh-CAT* mRNA expression levels were analyzed by GraphPad Prism 9.3.1 software (GraphPad Software, San Diego, CA, USA) following nonparametric one-way analysis of variance (ANOVA). Tukey’s post hoc test was performed to calculate statistically significant differences among different experimental tissues of Pacific abalone. Data from qRT-PCR are expressed as the mean ± SD. Differences were considered as significant at *p* < 0.05. GraphPad Prism 9.3.1 software was used to generate graphs. 

## 3. Results

### 3.1. Cloning and Bioinformatic Analysis of H. discus hannai Catalase (Hdh-CAT) Sequence

**Figure 1:**
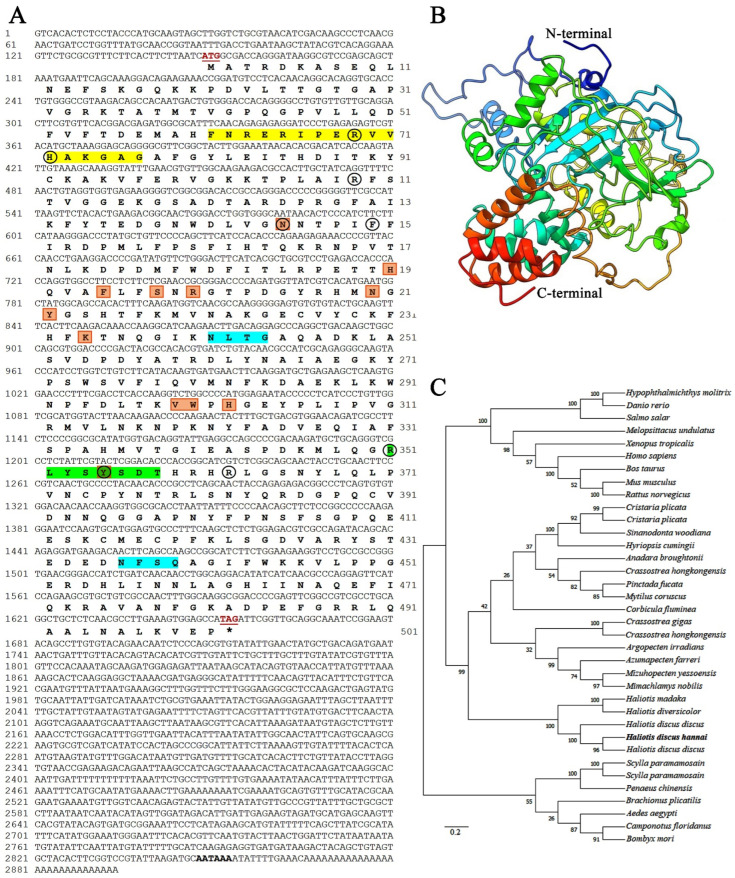
Sequence analysis of catalase (*Hdh-CAT*) in Pacific abalone, *H. discus hannai*. (**A**) The full-length nucleotide (GenBank: OK042347.1) and deduced amino acid sequences (GenBank: UFT26656.1). Numbers on the left and right columns indicate nucleotide and amino acid positions in the sequence, respectively. The initiation (ATG) and termination (TAG) codons of nucleotide sequence are marked as bold red color and underlined. The stop codon of nucleotide sequence was indicated using *. *Hdh-CAT* active site motif and heme-ligand signature motif are highlighted using yellow and light green colors, respectively. Heme-binding site residues are pointed using circle. Two putative *N*-glycosylation sites are indicated using light sky color. The putative NADPH binding residues are pointed using box. The polyadenylation signal (AATAAA) is indicated in boldface. (**B**) Three-dimensional (3D) homology modeling of Hdh-CAT. (**C**) Phylogenetic tree of catalase protein sequences from vertebrates and invertebrates. GenBank accession number of all sequences of phylogenetic tree are as follows: *Hypophthalmichthys molitrix* (ADJ67807.1), *Danio rerio* (NP_570987.2), *Salmo salar* (ACN11170.1), *Melopsittacus undulatus* (AAO72713.1), *Xenopus tropicalis* (AAH90377.1), *Homo sapiens* (NP_001743.1), *Bos taurus* (3NWL_A), *Mus musculus* (P24270.4), *Rattus norvegicus* (NP_036652.1), *Cristaria plicata* (ADM64337.1), *C. plicata* (AEL31245.1), *Sinanodonta woodiana* (AMO00631.1), *Hyriopsis cumingii* (ADL14588.1), *Anadara broughtonii* (ALZ42087.1), *Crassostrea hongkongensis* (ADZ76134.1), *Pinctada fucata* (ADW08700.1), *Mytilus coruscus* (AQY56552.1), *Corbicula fluminea* (APX42721.1), *C. gigas* (ABS18267.1), *C. hongkongensis* (ADZ93495.1), *Argopecten irradians* (ADD71945.1), *Azumapecten farreri* (ABI64115.1), *Mizuhopecten yessoensis* (AKV63251.1), *Mimachlamys nobilis* (AHX22599.1), *H. madaka* (ALU63753.1), *H. diversicolor* (AEP83810.1), *H. discus discus* (ABF67505.1), *H. discus hannai* (UFT26656.1), *H. discus discus* (ABQ60044.1, ABF67505.1), *Scylla paramamosain* (QNT61282.1), *S. paramamosain* (ACX46120.1), *Penaeus chinensis* (ABW82155.1), *Brachionus plicatilis* (BAH28837.1), *Aedes aegypti* (EAT34333.1), *Camponotus floridanus* (EFN66292.1), *Bombyx mori* (NP_001036912.1).

The complete sequence of *H. discus hannai* catalase was cloned from digestive gland samples by 5′-RACE and 3′-RACE PCR and named Hdh-CAT (Figure 1A). The full-length sequence of *Hdh-CAT* (GenBank: OK042347.1) was 2894 bp in length, including a 148 bp 5′-untranslated region (UTR) and a 1240-bp 3′-UTR with a canonical polyadenylation signal sequence (AATAAA) located 12 bp upstream of the poly-A tail. The open reading frame (ORF) of Hdh-CAT had a length of 1506 bp, encoding 501 deduced amino acids (GenBank: UFT26656.1). 

The molecular weight and theoretical isoelectric point (pI) of Hdh-CAT protein were predicted to be 56.46 KDa and 8.81, respectively. Glycine was the most abundant amino acid (7.8%), while tryptophan was the least abundant (1.2%) in the deduced protein sequence. Motif scan and conserved domain analysis revealed that Hdh-CAT has a catalase domain at position 25–410 amino acid residues with an E-value of 5.01E-280. The Hdh-CAT deduced amino acid sequence covered a highly conserved catalytic site motif, [F/I]-X-[R]-XXXX-[ER]-XX-[H]-XX-[G/A/S]-[G/A/S/T/F/Y]-[G/A/S/T], at the positions 61–77 (FNRERIPERVVHAKGAG). The proximal heme-ligand signature motif (RLYSYSDT) was detected at amino acid positions 351–358. Heme-binding site residues were identified at eight positions (R_69_, H_72_, R_109_, N_145_, F_150_, R_351_, Y_355_, and R_362_).

Additionally, the catalytic residue of histidine (H) at position 72 was identified as a proper binding, and peroxide reduction site. Three amidation sites were found at amino acid positions of 32–35 (VGRK), 100–103 (VGKK), and 486–489 (FGRR). Two N-glycosylation sites were found at amino acid positions of 241–244 (NLTG) and 436–439 (NFSQ). Four N-myristoylation sites were found at amino acid positions of 29–34 (GAPVGR), 114–119 (GGEKGS), 201–206 (GTPDGY), and 396–401 (GGAPNY). 

Phosphorylation site analysis showed six protein kinase C phosphorylation sites, [T/S]-X-[K/R], at amino acid positions of 122–124 (TAR), 164–166 (TQK), 184–186 (TLR), 198–200 (SNR), 216–218 (TFK), and 358–360 (THR). Casein kinase II phosphorylation site was detected at the positions of 122–125 (TARD), 338–341 (TGIE), 354–357 (SYSD), and 430–433 (STED). A tyrosine kinase phosphorylation site, [K/R]-XXX- [E/D]-XXX-[Y], was detected at amino acid positions 421–429 (KLSGDVARY). Catalase-related immune-responsive sites were found at amino acid positions 418–495 with an E-value of 1.5E-33.

Gene ontology (GO) term analysis predicts that Hdh-CAT had catalase activity with a heme-binding ability (Figure S1A) in molecular function (GO: 0003674), hydrogen peroxidase catabolic process (Figure S1B) in biological process (GO:0008150), and located in peroxisome (Figure S1C) of cellular component (GO:0005575).

Multiple alignments of catalase homologs from selected amino acid sequences of vertebrate and invertebrate are shown in Figure S2. Active site motif and heme-ligand signature motif were well conserved in aligned amino acid sequences of *H. discus discus*, *H. diversicolor*, *Danio rerio,* and *Homo sapiens*. Multiple alignment revealed 12 NADPH binding site residues (N_145_, H_191_, F_195_, S_198_, R_200_, N_210_, Y_212_, K_234_, V_299_, W_300_, H_302_, and Y_355_). Hdh-CAT shared the highest sequence identities (99.38%) with CAT of *H. discus discus*.

### 3.2. Homology Modeling of Hdh-CAT

Three-dimensional structures of *Hdh-CAT* exhibited four basic domains including an N-terminal domain, an eight-stranded β-barrel domain, a wrapping loop-formed connection domain, and a helical C-terminal domain (Figure 1B).

### 3.3. Phylogenetic Analysis

A phylogenetic tree was constructed using the neighbor-joining method to show the possible evolutionary linkage of *Hdh-CAT* with other catalase proteins. The phylogenetic tree showed three major groups where *Hdh-CAT* was positioned in the mollusk group (Figure 1C). *Hdh-CAT* was closely positioned with *H. discus discus* catalase. 

### 3.4. Tissue Distribution Analysis of Hdh-CAT

*Hdh-CAT* mRNA expression levels in different tissue samples are shown in Figure S3. The expression levels of *Hdh-CAT* mRNA were significantly higher in the digestive gland (DG) tissue samples than in other examined tissue samples.

### 3.5. Fluorescence In Situ Hybridization 

**Figure 2:**
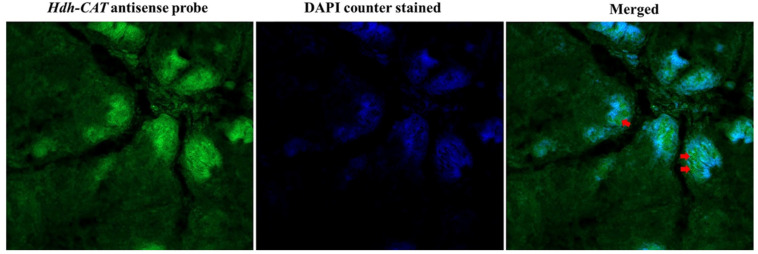
Confocal laser scanning microscopic observation after fluorescence in situ hybridization of *Hdh-CAT* mRNA in digestive gland tissue of Pacific abalone. The positive signals of *Hdh-CAT* mRNA were indicated using arrows. Scale bar: 50 μm.

Fluorescence *in situ* hybridization (FISH) revealed that *Hdh-CAT* was localized in the digestive tubules of the digestive gland of Pacific abalone. The antisense probe of FISH showed positive signals (green fluorescent) in the digestive tubules of the digestive gland (Figure 2). Blue fluorescent (DAPI-stained) counter-stained the positive signal of *Hdh-CAT*. Confocal laser scanning image confirmed that the *Hdh-CAT* localized in the Pacific abalone digestive gland (Figure 2).

### 3.6. Hdh-CAT mRNA Expression in Thermal Stressed Tissue Samples of Pacific Abalone

**Figure 3:**
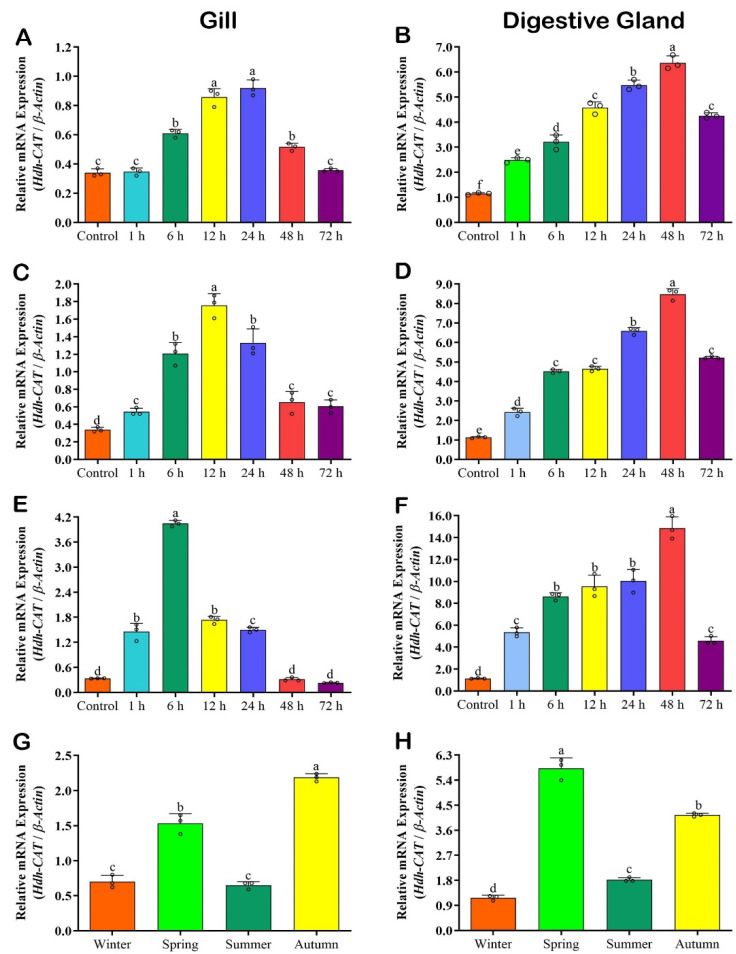
*Hdh-CAT* mRNA expression levels in thermal stressed and during elevated seasonal temperatures of Pacific abalone at different time points. (**A**) Cold-stressed (15 °C) gill samples. (**B**) Cold-stressed digestive gland (DG) samples. (**C**) Heat-stressed (25 °C) gill samples. (**D**) Heat-stressed (25 °C) DG samples. (**E**) Heat-stressed (30 °C) gill samples. (**F**) Heat-stressed (30 °C) DG samples. (**G**) *Hdh-CAT* mRNA expression level in gill during elevated seasonal temperatures. (**H**) *Hdh-CAT* mRNA expression level in DG during elevated seasonal temperatures. Different letters above the bars indicate significant differences (*p* < 0.05).

Expression levels of *Hdh-CAT* mRNA in gill and digestive gland (DG) tissues of thermal-stressed Pacific abalone are shown in Figure 3.

#### 3.6.1. *Hdh-CAT* mRNA Expression in Cold Stressed (15 °C) Samples 

The mRNA expression level of *Hdh-CAT* reached its peak at time points of 24 h in the gill (Figure 3A), and 48 h in the DG (Figure 3B) of Pacific abalone. 

#### 3.6.2. *Hdh-CAT* mRNA Expression in Heat Stressed (25 °C) Samples 

*Hdh-CAT* mRNA expression level was significantly highest at time points of 12 h in the gill (Figure 3C), and 48 h in the DG (Figure 3D). However, in gill samples, the expression level was neutralized at time points of 48 h to 72 h. 

#### 3.6.3. *Hdh-CAT* mRNA Expression in Heat Stressed (30 °C) Samples 

The mRNA expression level of *Hdh-CAT* in gill samples of heat-stressed Pacific abalone was significantly (*p* < 0.05) higher at time points of 6 h (Figure 3E). DG samples showed a significantly (*p* < 0.05) higher expression level of *Hdh-CAT* mRNA at time points of 48 h (Figure 3F). 

### 3.7. Hdh-CAT mRNA Expression in Seasonal Samples

*Hdh-CAT* mRNA expression levels in gill were significantly (*p* < 0.05) higher in autumn (Figure 3G) compared to other seasons. Whereas DG samples showed significantly (*p* < 0.05) higher *Hdh-CAT* mRNA expression levels in spring (Figure 3H). 

### 3.8. Hdh-CAT mRNA Expression in H_2_O_2_ Induced Tissue Samples of Pacific Abalone

**Figure 4:**
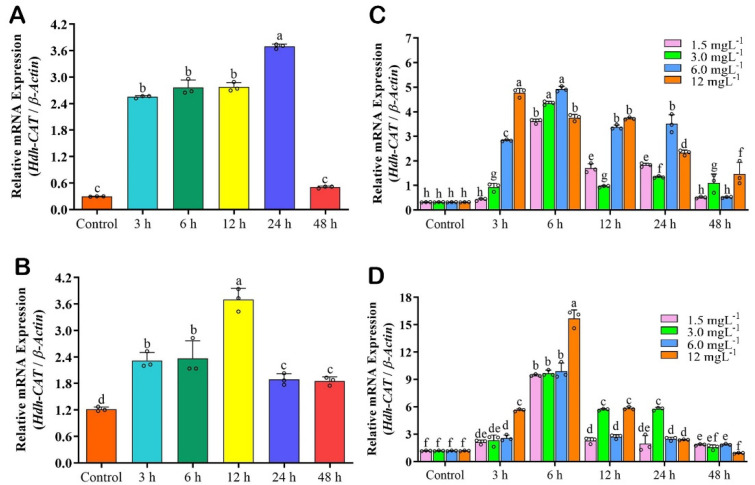
*Hdh-CAT* mRNA expression levels in H_2_O_2_ induced and cadmium (Cd) exposed (1.5 mgL^−1^, 3.0 mgL^−1^, 6.0 mgL^−1^, and 12.0 mgL^−1^) Pacific abalone at different time points. (**A**) *Hdh-CAT* mRNA expression level in gill of H_2_O_2_ induced Pacific abalone. (**B**) *Hdh-CAT* mRNA expression level in digestive gland (DG) of H_2_O_2_-induced Pacific abalone. (**C**) *Hdh-CAT* mRNA expression level in gill of Cd-exposed Pacific abalone. (**D**) *Hdh-CAT* mRNA expression level in DG of Cd-exposed Pacific abalone. Different letters above the bars indicate significant differences (*p* < 0.05).

The mRNA expression level of *Hdh-CAT* reached its peak at time points of 24 h in the gill (Figure 4A), and 12 h in the DG (Figure 4B) of H_2_O_2_-induced Pacific abalone. 

### 3.9. Hdh-CAT mRNA Expression in CdCl_2_ Exposed Tissues of Pacific Abalone

The expression levels of *Hdh-CAT* mRNA in different concentrations of CdCl_2_ exposed tissue samples are presented in Figure 4C,D. *Hdh-CAT* mRNA expression levels were significantly (*p* < 0.05) different in different concentrations of CdCl_2_ exposed gill and DG samples, compared to the control. In gill samples, higher *Hdh-CAT* mRNA expression levels were shown at the time points of 3 h in the 12 mgL^−1^ CdCl_2_, 6 h in the 3 mgL^−1^, and 6 mgL^−1^ CdCl_2_, respectively (Figure 4C). DG tissue samples showed significantly higher *Hdh-CAT* mRNA at the time points of 6 h in the 12 mgL^−1^ CdCl_2_-exposed abalone (Figure 4D). 

### 3.10. Hdh-CAT mRNA Expression in Starved Tissue Samples of Pacific Abalone

**Figure 5:**
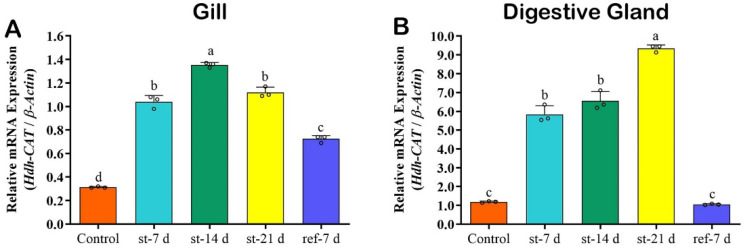
*Hdh-CAT* mRNA expression levels in starved and re-fed Pacific abalone at different time points. (**A**) *Hdh-CAT* mRNA expression level in gill of starved Pacific abalone. (**B**) *Hdh-CAT* mRNA expression level in DG of starved Pacific abalone. Different letters above the bars indicate significant differences (*p* < 0.05).

*Hdh-CAT* mRNA expression levels were significantly (*p* < 0.05) higher in Pacific abalone gill at 14 days after starvation (st-14 d) (Figure 5A). DG samples showed significantly (*p* < 0.05) higher *Hdh-CAT* mRNA expression levels at 21 days after starvation (st-21 d) (Figure 5A) of Pacific abalone. After re-feeding for seven days, *Hdh-CAT* mRNA expression levels in DG of starved samples were similar to those in control samples without starvation (Figure 5B).

### 3.11. Hdh-CAT mRNA Expression in Immune Challenged Tissues of Pacific Abalone

#### 3.11.1. *Hdh-CAT* Expression in *Vibrio parahaemolyticus* Challenged Samples

**Figure 6:**
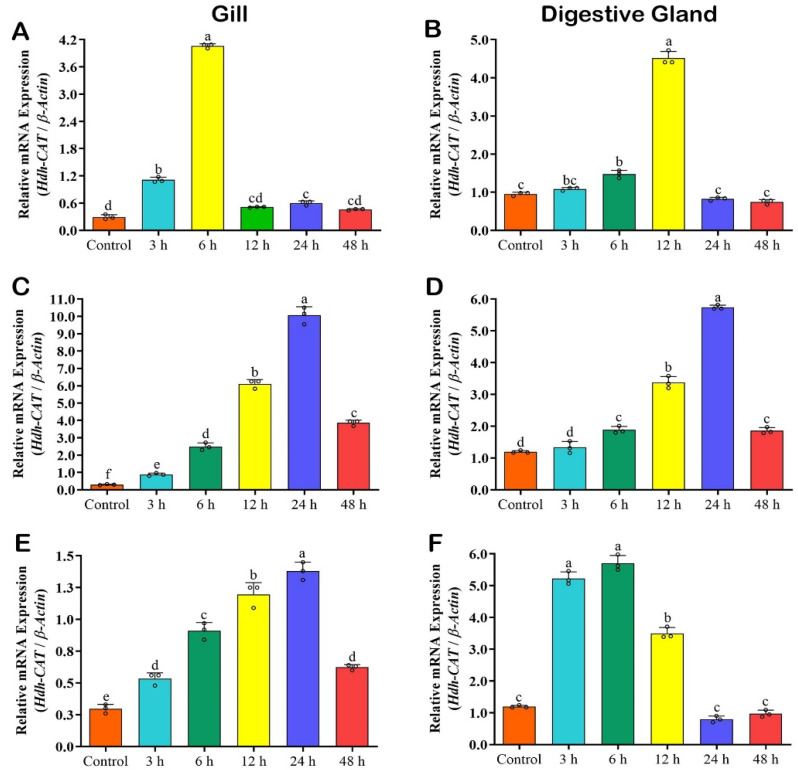
*Hdh-CAT* mRNA expression levels in immune-challenged Pacific abalone at different time points. (**A**) *V. parahaemolyticus*-challenged gill samples. (**B**) *V. parahaemolyticus*-challenged DG samples. (**C**) Lipopolysaccharides (LPS)-challenged gill (GIL) samples. (**D**) LPS-challenged digestive gland (DG) samples. (**E**) Polyinosinic–polycytidylic acid sodium salt (PIC)-challenged gill samples. (**F**) PIC-challenged DG samples. Different letters above the bars indicate significant differences (*p* < 0.05).

The mRNA expression level of *Hdh-CAT* reached its peak in the gill at 6 h after *V. parahaemolyticus* challenge (Figure 6A) and in the DG at 12 h after *V. parahaemolyticus* challenge (Figure 6B). Expression levels were stabilized in both tissue samples at 24 h and 48 h after *V. parahaemolyticus* challenge (Figure 6A,B).

#### 3.11.2. *Hdh-CAT* Expression in Lipopolysaccharides (LPS) Challenged Samples

The mRNA expression level of *Hdh-CAT* reached its peak at time points of 24 h in the gill (Figure 6C), and DG (Figure 6D) of LPS-challenged Pacific abalone. 

#### 3.11.3. *Hdh-CAT* Expression in Poly I:C Challenged Samples

The mRNA expression level of *Hdh-CAT* reached its peak at time points of 24 h in the gill (Figure 6E), and 6 h in the DG (Figure 6F) of PIC-challenged Pacific abalone. 

### 3.12. Hdh-CAT mRNA Expression in Testicular Developmental Stages

**Figure 7:**
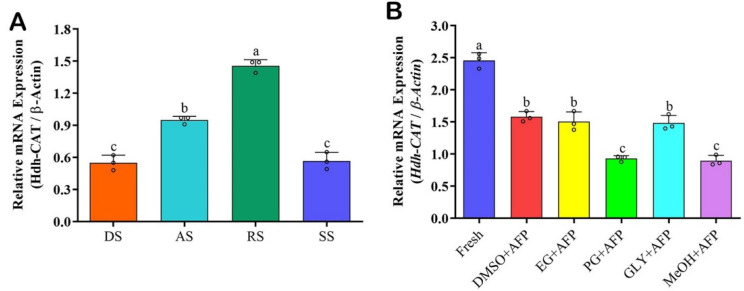
*Hdh-CAT* mRNA expression levels at different testicular developmental stages and in cryopreserved sperm samples of Pacific abalone. (**A**) *Hdh-CAT* mRNA expression level in different testicular developmental stages. (**B**) *Hdh-CAT* mRNA expression levels at cryopreserved sperm samples. DS, degenerative stage; AS, active stage; RS, ripening stage; SS, spent stage; DMSO + AFP, 8% dimethyl sulfoxide + antifreeze protein; EG + AFP, 8% ethylene glycol + antifreeze protein; PG + AFP, 6% propylene glycol + antifreeze protein; GLY + AFP, 2% glycerol + antifreeze protein; and MeOH + AFP, 2% methanol + antifreeze protein. Different letters above the bars indicate significant differences (*p* < 0.05).

*Hdh-CAT* mRNA expression levels during different testicular developmental stages are given in Figure 7A. Ripening stage showed significantly (*p* < 0.05) higher *Hdh-CAT* mRNA expression levels compared to other stages (Figure 7A).

### 3.13. Hdh-CAT mRNA Expression in Cryopreserved Sperm

*Hdh-CAT* mRNA expression levels in fresh sperm and different types of cryopreserved sperm samples are shown in Figure 7B. *Hdh-CAT* mRNA expression levels in cryopreserved sperm samples were significantly lower than those in fresh sperm (Figure 7B). 

### 3.14. Hdh-CAT mRNA Expression in Embryonic and Larval Developmental Stages 

**Figure 8:**
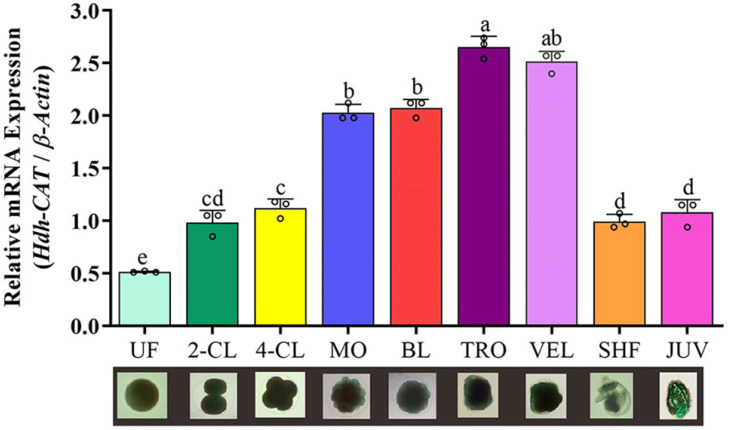
*Hdh-CAT* mRNA expression levels during metamorphosis of Pacific abalone. UF; unfertilized egg, 2-CL; 2-cell, 4-CL; 4-cell, MO; morula, BL; blastula, TRO; trochophore larvae, VEL; veliger larvae, SHF; shell formed larvae, JUV; juvenile. Different letters above the bars indicate significant differences (*p* < 0.05).

*Hdh-CAT* mRNA expression levels during embryogenesis are shown in Figure 8. Blastula (BL) and trochophore (TRO) larvae showed significantly (*p* < 0.05) higher *Hdh-CAT* mRNA expression levels compared to other stages. 

### 3.15. Catalase (CAT) Activity in the Hemolymph 

**Figure 9:**
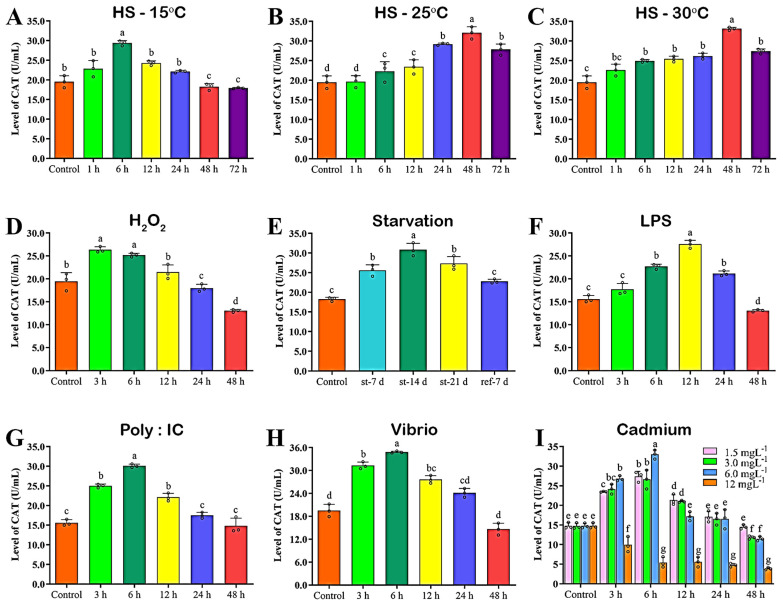
Catalase (CAT) activity in the different types of stress and immune-challenged plasma of Pacific abalone. (**A**) CAT activity in cold-stressed (15 °C) abalone, (**B**) CAT activity in heat-stressed (25 °C) abalone, (**C**) CAT activity in heat-stressed (30 °C) abalone, (**D**) CAT activity in H_2_O_2_-induced abalone, (**E**) CAT activity in starved abalone, (**F**) CAT activity in LPS-challenged abalone, (**G**) CAT activity in poly: (IC)-challenged abalone, (**H**) CAT activity in *V. parahaemolyticus*-challenged abalone, (**I**) CAT activity in cadmium-exposed abalone. Significantly different levels (*p* < 0.05) are denoted by different letters.

CAT activities in hemolymph samples of cold (15 °C), heat-stressed (25 °C and 30 °C), H_2_O_2_-induced, starved, immune-challenged, and Cd-exposed abalone are summarized in Figure 9. Cold-stressed abalone showed significantly (*p* < 0.05) higher CAT activity at 6 h after cold stress (Figure 9A), whereas abalone with heat stress (25 °C and 30 °C) showed higher CAT activities at 48 h after heat stress (Figure 9B,C). H_2_O_2_-induced abalone showed higher CAT activity at the time point 3 h (Figure 9D), significantly similar to the time point of 6 h post-induction. Starved abalone showed significantly higher CAT activities at st-14 d (Figure 9E). However, re-fed (ref-7 d) abalone showed similar (*p* > 0.05) CAT activity to the control. LPS-challenged abalone showed significantly (*p* < 0.05) higher CAT activity at 12 h after LPS challenge (Figure 9F). PIC (Figure 9G) and *V. parahaemolyticus* (Figure 9H)-challenged abalone showed significantly higher CAT activity at 6 h after challenge. Cd exposure increased CAT activity in a dose-dependent manner (Figure 9I). After exposure to 6 mgL^−1^ of Cd, CAT activity was significantly higher at 6 h after exposure (Figure 9I).

### 3.16. ROS in DG Tissue Samples of Pacific Abalone

**Figure 10:**
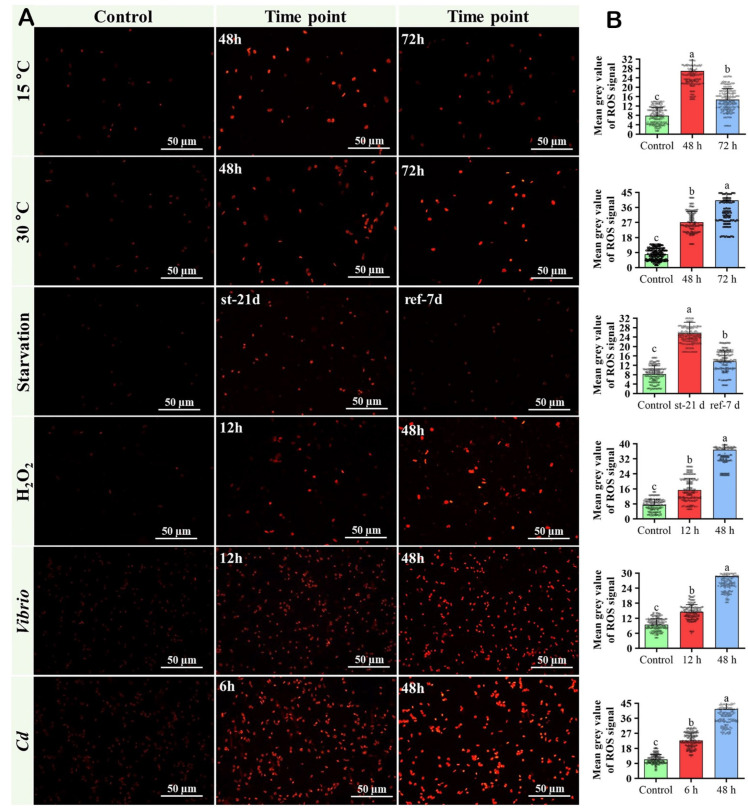
Detection of reactive oxygen species (ROS) in thermal-stressed, starved, H_2_O_2_-induced, bacterial-challenged, and Cd-exposed Pacific abalone. (**A**) Fluorescent images of dihydroethidium (DHE) stained digestive gland tissue samples. (**B**) Level of ROS production was analyzed by measuring mean grey values of DHE signal. Significantly different levels (*p* < 0.05) are denoted by different letters.

The results of ROS production in response to thermal stress (15 °C and 30 °C), starvation, H_2_O_2_ induction, *Vibrio* challenged, and Cd-exposed toxicity are presented in Figure 10. Heat-induced (30 °C) abalone showed higher ROS production at 72 h (Figure 10A,B). H_2_O_2_-induced, Vibrio-challenged, and Cd-exposed abalone showed higher ROS production at 48 h (Figure 10A,B). However, re-feed abalone showed reduced ROS production (Figure 10A,B).

## 4. Discussion

The aim of the present study was to isolate catalase and detect its molecular regulation in stress physiology, innate immunity, testicular development, elevated seasonal temperature, metamorphosis, and cryopreserved sperm of Pacific abalone. The full-length cDNA of Pacific abalone catalase (*Hdh-CAT*) was isolated for the first time, and its expression in digestive gland tissue was characterized. The architecture of *Hdh-CAT* showed key features of a catalase gene, including a proximal heme-ligand signature motif, a proximal active site signature, and heme-binding site residues. These features are common in most catalases [3]. 

Fluorescence *in situ* hybridization confirmed the localization of *Hdh-CAT* mRNA in the digestive tubules of the digestive gland (DG). Previous studies have reported that CAT is immunolocalized in the digestive tubules of different mollusks including oyster, and mussel [52]. CAT is also immunolocalized in the digestive tubules of crab, and the liver of mullet [52]. Molluscan DG has combined functions of the liver, pancreas, and intestine of vertebrates [53]. The liver is the predominant source of peroxisomes where CAT is exclusively located [54,55]. Tissue distribution analysis also revealed that *Hdh-CAT* mRNA was highly expressed in DG tissue samples, consistent with findings of CAT in disk abalone [3]. 

Temperature is a vital abiotic factor that can significantly affect the physiology of marine organisms [21]. In recent decades, seawater temperature has been rising with the acceleration of climate change [21,56]. Temperature changes are known to influence ROS production and activate antioxidant enzymes in mollusk [57]. Marine invertebrates have strong antioxidant defense systems including CAT to normalize ROS accumulation and physiological function [58]. An elevated activity of CAT is essential to counteract thermal stress-induced oxidative damage [59]. In the present study, induced CAT activity and higher *Hdh-CAT* mRNA expression levels were observed in response to thermal stress. Similar findings have been previously reported for CAT in scallop [4], ark shell [21], Mediterranean mussel [60], and Pacific oyster [61]. The present study also found elevated ROS levels in thermal-stressed abalone, consistent with previous findings of American oyster [31]. It has been reported that both heat and cold stress lead to excessive ROS production [13]. Present findings suggest that thermal stress can increase the CAT activity of Pacific abalone which may have a relationship with ROS production since it has been described that the overproduction of ROS is associated with the dysfunction of the antioxidant defense system. The antioxidant defense system consists of five vital antioxidant genes including catalase, Cu/Zn-superoxide dismutase, manganese superoxide dismutase, glutathione peroxidase, and glutathione reductase [62]. 

H_2_O_2_ is ubiquitously distributed in the surface seawater [63]. H_2_O_2_ can affects the survivability, growth, and metabolism of aquatic organisms [64]. In the present study, H_2_O_2_-injected Pacific abalone showed upregulated *Hdh-CAT* mRNA expression in gill and DG. Similar phenomena have been reported in *H. discus discus* [3]. Induced CAT activity was detected in the hemolymph of H_2_O_2_-injected Pacific abalone. A lower concentration of CAT could not eliminate H_2_O_2_ which might accumulate excessive H_2_O_2_. Accumulated H_2_O_2_ might accelerate the downregulation of CAT [15]. The fluorescence technique detected higher ROS in DG at 48 h after H_2_O_2_ induction, which might be the reason for the lower CAT mRNA abundance and activity. Similar phenomena have been previously reported for CAT in American oyster [31]. Present findings suggest that Hdh-CAT might play a role in the antioxidant defensive mechanism against ROS generation induced by H_2_O_2_. Another antioxidant, SOD, has been previously recommended as an antioxidant defender against H_2_O_2_-induced damage in Pacific abalone [4]. 

Cd exposure increased *Hdh-CAT* mRNA expression levels in the gill and DG of Pacific abalone in a dose-dependent manner. Similar findings have been previously reported for CAT in the gill of Pacific oyster after Cd exposure [65]. Higher CAT mRNA expression levels could protect Pacific oyster against Cd exposure-induced oxidative stress [65]. The present study detected higher ROS in Cd-exposed DG using a fluorescent technique, although induced ROS production in Cd-exposed tissue samples of abalone has been previously reported with a colorimetric method [66]. In this study, hemolymph of Cd-exposed abalone showed induced CAT activity. Similar findings have been previously reported for marine invertebrate after exposure to toxic chemicals [67]. Induced H_2_O_2_ activity in the hemolymph of Cd-exposed Pacific oyster has been reported previously [65] since CAT is a main scavenger of H_2_O_2_. Taken together, present findings hypothesize that CAT could protect Pacific abalone against ROS induced by exposure to Cd, a heavy metal pollutant.

The present study showed higher *Hdh-CAT* mRNA expression levels in gill at two weeks and in DG of abalone at three weeks of starved abalone compared to the control. After two weeks of starvation, the gill sample showed gradually decreased *Hdh-CAT* mRNA expression levels in prolonged periods and re-feeding. However, DG showed gradually increased *Hdh-CAT* mRNA expression levels until three weeks of starvation. The re-fed sample showed an expression level of *Hdh-CAT* mRNA similar to the control. Similar phenomena have previously been reported from fish [68,69]. Starvation stimulates oxidative stress-oriented energy deficiencies [68]. When the starvation period is prolonged, cells produce ROS and gradually accumulate. In this circumstance, stored antioxidants cannot be supplied in vivo resulting in gradually decreased antioxidant capacity [23]. The present study also reported similar phenomena in the case of ROS production.

Gill and DG are important immune organs of Pacific abalone [44]. In this study, *V. parahaemolyticus* influenced the mRNA expression level of *Hdh-CAT* in gill and DG. Gill showed an earlier response (6 h) than DG (12 h) against *Vibrio* by showing higher *Hdh-CAT* mRNA abundance. A possible explanation for this time variation in different tissues might be the species- and tissue-specific nature of pathogen. *Vibrio*-infected abalone produced a significant amount of ROS; higher CAT mRNA might eliminate the excessive ROS generation. In mollusk, antioxidant response and ROS production show wide variations depending on tissue type, pathogen, and host [28]. *Vibrio* challenges also induced mRNA expression of catalase in the digestive gland and gill of *Mytilus galloprovincialis* and *H. discus discus* [28,70]. The present findings suggest that *Hdh-CAT* might play a role in the innate immune response of Pacific abalone against *V. parahaemolyticus*.

Lipopolysaccharide (LPS) is a well-recognized pathogen-associated immune stimulant [44]. In the present study, *Hdh-CAT* mRNA expression levels were quantified from gill and DG in response to LPS challenge at different time points. Upregulated *Hdh-CAT* mRNA expression was determined in both the gill and DG of LPS-challenged Pacific abalone. The expression of superoxide dismutase (SOD), an antioxidant gene, has been previously reported to be upregulated in the gill and DG of Pacific abalone [44]. CAT is activated immediately after the activation of SOD in the oxidative defense system. LPS challenge can also upregulate CAT expression level in the hepatopancreases or DG of *Scylla paramamosain* [71]. 

Poly I:C (PIC) is a synthetic viral mimic extensively used in viral infection experiments [72]. In the present study, PIC induced *Hdh-CAT* mRNA expression levels in the gill and DG tissues of Pacific abalone. PIC can also induce mRNA expression of CAT in the liver (DG) of *Bostrychus sinensis* [73]. Upregulated mRNA expression of CAT in viral challenged gill of *H. discus discus* has been previously reported [28]. Results of the present study suggest that PIC challenge might activate the immune response in DG more than in gill since DG is a key metabolic organ of mollusk [74]. 

Sperm have multiple antioxidants including CAT which can be altered during cryopreservation [75]. The present study found that cryopreserved sperm had significantly lower *Hdh-CAT* mRNA abundance than fresh sperm. Similar findings have been previously reported for cryopreserved rooster sperm [35]. Results of the present study suggest that *Hdh-CAT* might be considered as a biomarker of cryopreserved sperm of Pacific abalone.

The present study showed higher *Hdh-CAT* mRNA expression levels in trochophore (TRO) and veliger (VEL) larvae stages of embryogenesis. In abalone, trochophore larvae are considered as hatching steps and veliger larvae are considered as hatched larvae of embryogenesis [76]. Similar findings have been previously observed in Atlantic bluefin tuna [32] and Seabass [77]. The present findings suggest that *Hdh-CAT* might be involved in hatching succession in the embryogenesis process of Pacific abalone.

## 5. Conclusions

*Hdh-CAT* was for the first time cloned from the digestive gland of Pacific abalone, *H. discus hannai*. *Hdh-CAT* was localized in the digestive tubules of the digestive gland. Catalase activity and mRNA expression analysis indicated that *Hdh-CAT* might regulate the antioxidant defense system against thermal stress, viral infection, bacterial infection, starvation, and cadmium-induced toxicity. *Hdh-CAT* might have a potential role in testicular development and metamorphosis. Our findings also suggest that *Hdh-CAT* may have a defensive role against excessive ROS production. Expression levels of *Hdh-CAT* in cryopreserved sperm suggest that *Hdh-CAT* might be used as an indicator of cryotolerance of Pacific abalone sperm. Taken together, the present findings suggest that Hdh-CAT might be used as a stress and toxicity indicator.

## Data Availability

All data generated in this study are included in this published article and its supplementary information files.

## Supplementary Materials

The following supporting information can be downloaded at: https://www.mdpi.com/article/10.3390/antiox12010109/s1, Figure S1: Functional activity analysis of Hdh-CAT amino acid sequence of Pacific abalone, *H. discus hannai*. (**A**) Molecular function, (**B**) Biological process, (**C**) Cellular component; Figure S2: Multiple sequence alignment of deduced amino acid sequences of different catalase (CAT) from *H. discus hannai* (UFT26656.1), *H. discus discus* (ABQ60044.1), *H. madaka* (ALU63753.1), *H. diversicolor* (AEP83810.1), *Danio rerio* (NP_570987.2), and *Homo sapiens* (NP_001743.1). CAT active site motif and heme-ligand binding site motif are indicated by blacked lined box. Conserved NADPH binding site residues are pointed using a symbol on the top of each residue; Figure S3: Expression of *Hdh-CAT* mRNA in different tissue of Pacific abalone, *Haliotis discus hannai*. GIL; gill, DG; digestive gland, MNT; mantle, MUS; muscle, TE; testis, OV; ovary, HCY; hemocyte, HRT; heart, PPG; pleuropedal ganglion, CG; cerebral ganglion. Different letters above the bar indicate significance difference (*p* < 0.05); Table S1: List of primers used for cDNA cloning, tissue distribution, and expression analysis of *Hdh-CAT* in Pacific abalone.
