# Supplementary material for: Molecular Cloning and Functional Characterization of Catalase in Stress Physiology, Innate Immunity, Testicular Development, Metamorphosis, and Cryopreserved Sperm of Pacific Abalone
Source: Antioxidants (Basel). 2023 Jan 1;12(1):109. doi: 10.3390/antiox12010109 (PMC9854591; doi:10.3390/antiox12010109)
Supplement: Supplementary file 1 [file antioxidants-12-00109-s001.zip › antioxidants-2081488-supplementary.pdf]

[*Supplementary file*]

Molecular Cloning and Functional Characterization of Catalase in Stress Physiology, Innate Immunity, Testicular Development, Metamorphosis, and Cryopreserved Sperm of Pacific Abalone

Table S1. List of primers used for cDNA cloning, tissue distribution, and expression analysis of *Hdh-CAT* in Pacific abalone.

Primer name	Nucleotide sequence (5′---3′)	Purpose
CAT-Fw	ATGGAGTGCCCTTTCAAGC	RT-PCR
CAR-Rv	CTATGGCTCCACTTTCAAGG	
Hdh-CAT-3′ RACE	GATTACGCCAAGCTTGTGCCCTTTCAAGCTCTCTGGAGACG	RACE PCR
Hdh-CAT-5′ RACE	GATTACGCCAAGCTTCTCTGTGCTGTATCTGGCGACGTCTC	
Universal primer (short)	CTAATACGACTCACTATAGGGC	
Universal primer (long)	CTAATACGACTCACTATAGGGCAAGCAGTGGTATCAACGCAGAGT	
Hdh-CAT-Fw	CTGAGAGAGTCGTACATGC	qRT-PCR
Hdh-CAT-Rv	CCTTCTCACCACCTACAGTT	
β-Actin-Fw	CCGTGAAAAGATGACCCAGA	
β-Actin-Rv	TACGACCGGAAGCGTACAGA	
Hdh-CAT-Sense	CATGGCGACCAGGGATAAG	FISH
Hdh-CAT-Antisense	CCTTGGTTTGTCTTGAAGTGA	
Oligo dT (OdT)	GGCCACGCGTCGACTAGTACTTTTTTTTTTTTTTTTT	cDNA synthesis
Oligo dT adapter (AP)	GGCCACGCGTCGACTAGTAC	

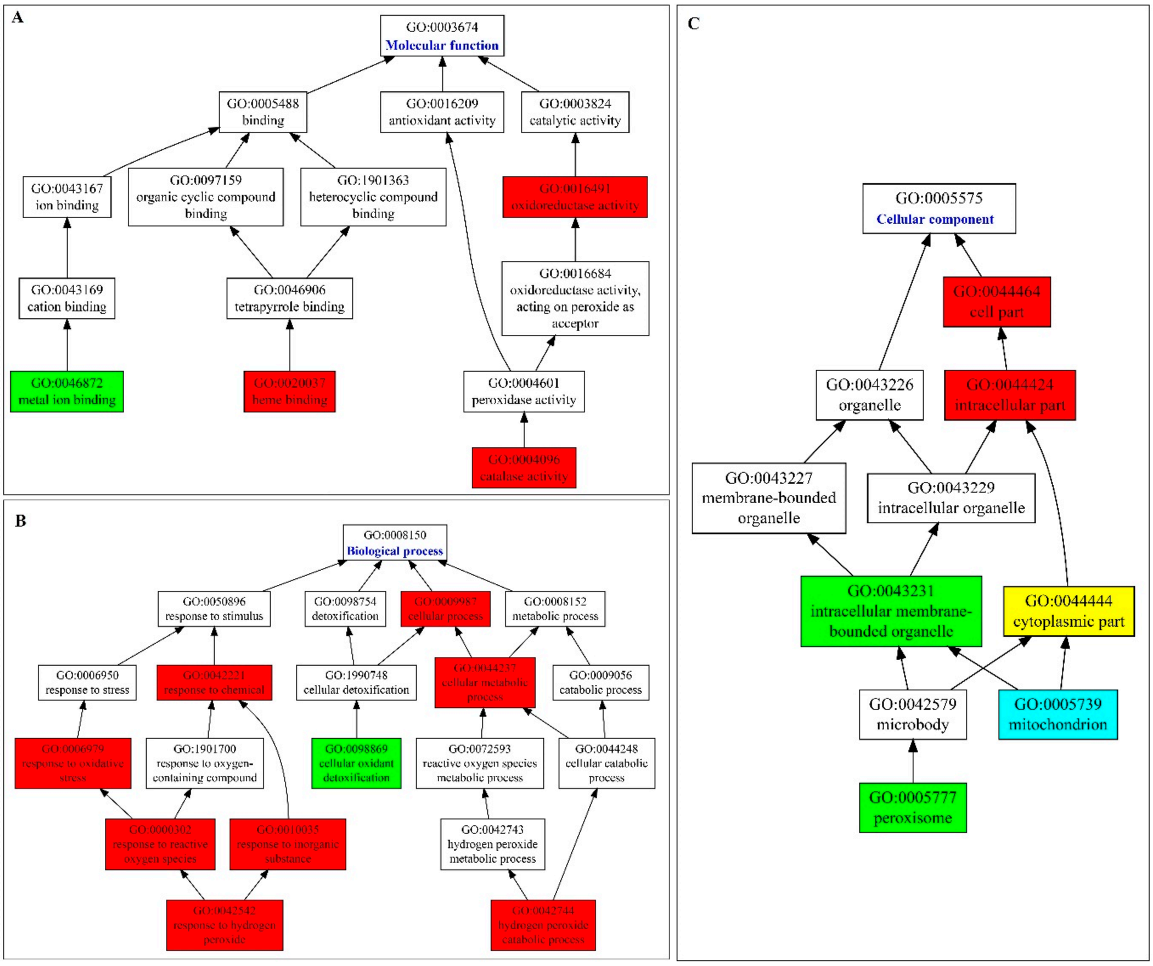

Figure S1. Functional activity analysis of Hdh-CAT amino acid sequence of Pacific abalone, *H. discus hannai*. (**A**) Molecular function, (**B**) Biological process, (**C**) Cellular component.

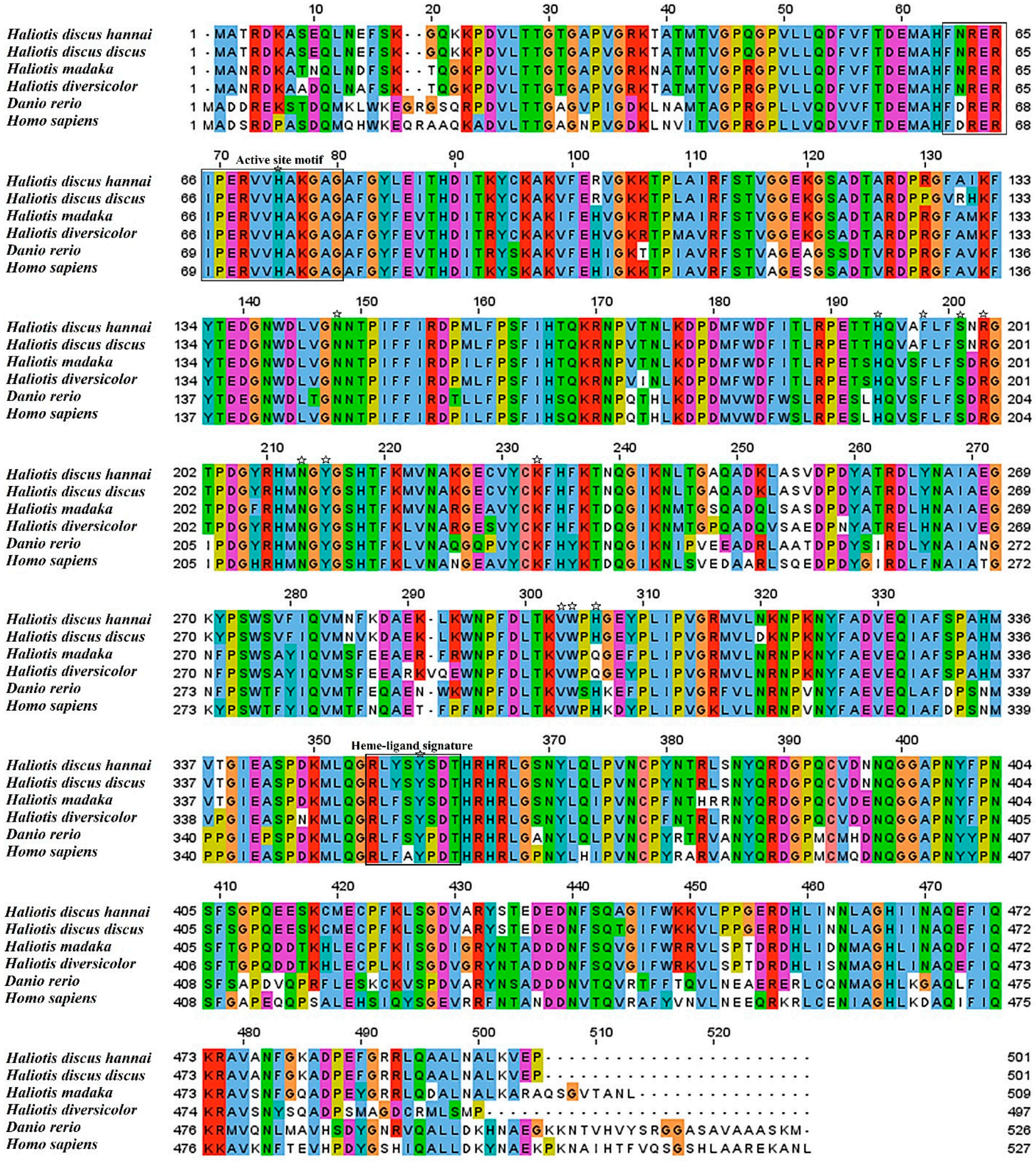

Figure S2. Multiple sequence alignment of deduced amino acid sequences of different catalase (CAT) from *H. discus hannai* (UFT26656.1), *H. discus discus* (ABQ60044.1), *H. madaka* (ALU63753.1), *H. diversicolor* (AEP83810.1), *Danio rerio* (NP_570987.2), and *Homo sapiens* (NP_001743.1). CAT active site motif and heme-ligand binding site motif are indicated by blacked lined box. Conserved NADPH binding site residues are pointed using a symbol on the top of each residue.

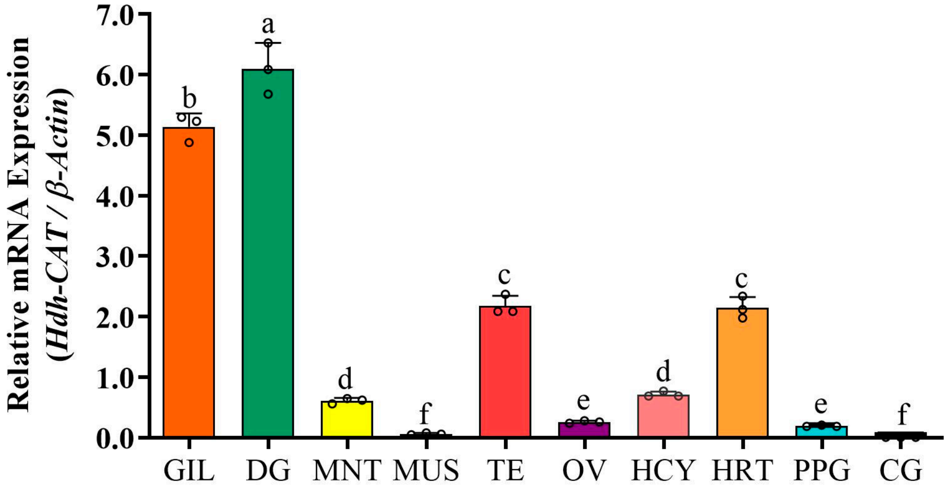

Figure S3. Expression of *Hdh-CAT* mRNA in different tissue of Pacific abalone, *Haliotis discus hannai*. GIL; gill, DG; digestive gland, MNT; mantle, MUS; muscle, TE; testis, OV; ovary, HCY; hemocyte, HRT; heart, PPG; pleuropedal ganglion, CG; cerebral ganglion. Different letters above the bar indicate significance difference ($p < 0.05$).
